# Supplementary material for: Comparative Effectiveness of Apixaban and Rivaroxaban Lead-in Dosing in VTE Treatment: Observational Multicenter Real-World Study
Source: J Clin Med. 2022 Dec 27;12(1):199. doi: 10.3390/jcm12010199 (PMC9821121; doi:10.3390/jcm12010199)
Supplement: Supplementary file 1 [file jcm-12-00199-s001.zip › jcm-2092344-supplementary.pdf]

Supplementary Table S1. Sub-group analysis of outcomes during hospitalization and up to 90 days after the VTE event in patients who received parenteral versus no parenteral anticoagulant in the recommended group.

Patient characteristic	Overall	Recommended lead-in		*p*-value
		Parenteral	No parenteral	
**Overall number of patients**	296	198	98	
**rVTE event**				
During Hospitalization	2 (0.7)	0 (0.0)	2 (2)	0.109
Within 30 days*	2 (0.7)	2 (1)	0 (0.0)	1.000
Cumulative within 90 days†	2 (0.7)	2 (1)	0 (0.0)	1.000
Patients with at least one rVTE within 90 days‡	4 (1.4)	2 (1)	2 (2)	0.602
**MB event**				
During Hospitalization	11 (3.7)	7 (3.5)	4 (4.1)	0.757
Within 30 days*	4 (1.4)	3 (1.5)	1 (1)	1.000
Cumulative within 90 days†	5 (1.7)	4 (2)	1 (1)	1.000
Patients with at least one MB within 90 days‡	14 (4.7)	9 (4.5)	5 (5.1)	0.780
**CRNMB event**				
During Hospitalization	9 (3.0)	8 (4)	1 (1)	0.083
Within 30 days*	23 (7.8)	18 (9.1)	5 (5.1)	0.228
Cumulative within 90 days†	23 (7.8)	18 (9.1)	5 (5.1)	0.228
Patients with at least one CRNMB within 90 days‡	29 (9.8)	24 (12.1)	5 (5.1)	0.056
**Rehospitalization§**				
Within 30 days*	7 (2.4)	7 (3.5)	0 (0.0)	0.099
Cumulative within 90 days†	13 (4.4)	12 (6.1)	1 (1)	0.067
**Death during hospitalization**	0 (0.0)	0 (0.0)	0 (0.0)	---

Results are presented as frequency (percentage).

p-values are from the chi-square or fisher-exact test.

Abbreviations: rVTE: recurrent venous thromboembolism; MB: major bleeding; CRNMB: clinically relevant non-major bleeding

* Within 30 days: 30 days from the indexed event date (excluding hospitalization days)

† Within 90 days: 90 days from the indexed event date (excluding hospitalization days)

‡ Cumulative number of patients with the outcome within 90 days, including events occurring during index hospitalization.

§ Rehospitalization due to VTE-related causes (recurrence, deterioration, or bleeding)

Note: Two patients in the recommended group who received parenteral therapy had two MB events each, and two patients had two CRNMB events each. with one patient in the recommended group who did not receive parenteral therapy had two CRNMB events during the 90 days of follow-up after the indexed VTE event, including events that occurred during the index hospitalization.
